# Notes and Comments

**Published:** 1899-09

**Authors:** 


### Notes and Comments.[1]

THERAPEUTIC CONSERVATISM.—*Merck's Archives* says, editorially, that a certain degree of conservatism in our attitude towards innovations, whether in therapeutics or anything else, is essential to safety. This is quite true, but when the conservative instinct causes us to refuse to examine new things, and, even when tested by others and declared good, keeps us from trying them under any circumstances, it becomes a dangerous obstruction. Justice to himself, to his patients, and to the race demands of every physician or dentist that he constantly seek to employ the very best remedies known, so that he may reach the maximum of cures. Unless one keeps informed of the discoveries in his profession, he cannot expect to reach this mark.

MOULDINE is made, according to Dr. Chupein, in *Dental Office and Laboratory*, by incorporating glycerin with finely powdered Fuller's earth. The glycerin is added a little at a time while the earth is in a mortar, the incorporation being effected by grinding with a pestle. It should be made into a mass the consistency of putty. In winter it gets quite hard and stiff, but it may be brought to its proper working consistency by heating it and working in, while warmed, a little more glycerin.

[1] The assistant editor solicits contributions for this department,—new methods, new remedies and formulas, or any short practical note which may prove of value to the practitioner or student. Address 1338 Walnut Street, Philadelphia.

Treatment of Carbolic Acid Poisoning.—The *Medical News* reports a case where a young boy of sixteen years had taken one and a half ounces of carbolic acid, the physician seeing him in thirty minutes from the time he swallowed it. He was in a limp and comatose state, the pulse being imperceptible. A pint of cream was at once poured into the stomach, which was kneaded in order to mix thoroughly the cream and the carbolic acid. Dry heat and friction were applied to the legs and arms. In two or three hours consciousness returned. The administration of cream and unskimmed milk was continued at short intervals for several hours. The patient entirely recovered in two days.

---

Removal of Iodine and Nitrate of Silver Stains.—Replying to inquiry for a method of removing the stain of iodine and nitrate of silver, we offer the following: To remove the stain of iodine from the skin or clothing the hyposulphite of soda acts very nicely; or it may be readily removed by the application of ammonia. If it happen to be on the lips of the patient, a weak solution should be used to bathe the spot. Nitrate of silver stain may be removed by first painting the spot with iodine, following the same with the application of ammonia.

---

Time Limit in Professional Work.—In reference to reading and other allied work, practitioners of dentistry seem to be divided into two classes: First, those who read several journals, buy and read the new books, and otherwise take time to improve themselves and keep abreast with the advance of their profession. These are usually the busy men. The second class claim it is not worth while to subscribe to more than one or two journals, as they seldom get time to look them over, and sometimes do not even take the wrapper off; they seldom buy a book and "have not time" to attend dental meetings. These constitute too large a portion of our members, and we have but to look about us to see that they are not the busy men of the profession; that is, from the general acceptance of the term. The *Lancet-Clinic* very tritely says,—

"Two things are never realized by the man who hasn't time; one is that there are exactly sixty minutes in every hour, and the other is like it, in which he fails to understand in its true bearings that there are neither more nor less than one hundred cents in every dollar. The busiest men attend the national, State, and local societies, and they are not disconcerted by pressing business engagements, a reason for which is found in the fact that they manage their business and do not allow their business to manage them."

---

Is the Enamel a Vital Tissue?—Dr. R. R. Andrews, of Cambridge, with Drs. Williams, Black, and others, claims that after the enamel is once formed there is no organic matter in it, and that it has no vitality. Dr. Andrews, in writing upon the subject, says,—

"I believe that enamel, fully formed, is nothing more nor less than a coat of mail, supplied by nature to protect the dentine and subserve the processes of mastication, and I doubt if it be possible, in perfectly formed human enamel, to find so much as a trace of organic matter within the substance of the enamel itself, or at its surface. Near its junction with the dentine there are nearly always found slight prolongations of the organic tissue, from the dentine, within its substance, and those are numerous enough at this point to account for the one to three per cent. of organic matter found in enamel on analysis.

"The enamel is lifeless; there is absolutely no difference to be found by the microscope, in the appearance of the enamel of a live tooth, from that of a tooth that has been a long time dead."

